# From phenotype to mechanism: Prenatal spectrum of *NKAP* mutation‐related disorder and its pathogenesis inducing congenital heart disease

**DOI:** 10.1111/jcmm.18305

**Published:** 2024-04-22

**Authors:** Xiayuan Xu, Chengcheng Gao, Fenglei Ye, Aohui Peng, Jianbo Xu, Keqin Jin, Jun Zhang, Yun Ye, Yanfen Yang, Xuan Zhang, Shuangshuang Shen, Fan Jin

**Affiliations:** ^1^ Department of Clinical Laboratory Jinhua Maternal and Child Health Care Hospital Jinhua Zhejiang China; ^2^ Key Laboratory of Reproductive Genetics (Ministry of Education) and Department of Reproductive Endocrinology Women's Hospital, School of Medicine Zhejiang University Hangzhou Zhejiang China; ^3^ Key Laboratory of Digital Technology in Medical Diagnostics of Zhejiang Province Dian Diagnostics Group Co., Ltd. Hangzhou Zhejiang China; ^4^ Department of Obstetrics and Gynecology Lishui Maternal and Child Health Care Hospital Lishui Zhejiang China; ^5^ College of Life Sciences Zhejiang Normal University Jinhua Zhejiang China; ^6^ Prenatal Diagnosis Center Jinhua Maternal and Child Health Care Hospital Jinhua Zhejiang China; ^7^ Department of Ultrasonography Jinhua Maternal and Child Health Care Hospital Jinhua Zhejiang China

**Keywords:** cardiac development, congenital heart defect, MRXSHD, *NKAP*, pathogenic mechanism, prenatal diagnosis

## Abstract

*NKAP* mutations are associated with Hackmann‐Di Donato‐type X‐linked syndromic intellectual developmental disorder (MRXSHD, MIM: #301039). Here, we elucidate the potential prenatal manifestation of *NKAP* mutation‐associated disorder for the first time, alongside revealing the relationship between *NKAP* mutations and congenital heart defect (CHD) in the Chinese population. An *NKAP* mutation (NM_024528.4: c.988C>T, p.Arg330Cys) was identified in two foetuses presenting with CHD. Subsequent mechanistic exploration revealed a marked downregulation of *NKAP* transcription within HEK293T cells transfected with *NKAP* p.R330C. However, no significant change was observed at the protein level. Moreover, the mutation led to a dysregulation in the transcription of genes associated with cardiac morphogenesis, such as *DHRS3*, *DNAH11* and *JAG1*. Additionally, our research determined that *NKAP* p.R330C affected Nkap protein intra‐nuclear distribution, and binding with Hdac3. Summarily, our study strengthens *NKAP* mutations as a cause of CHD and prompts the reclassification of *NKAP* p.R330C as likely pathogenic, thereby establishing a prospective prenatal phenotypic spectrum that provides new insight into the prenatal diagnosis of CHD. Our findings also provide evidence of *NKAP* p.R330C pathogenicity and demonstrate the potential mechanism by which p.R330C dysregulates cardiac developmental gene transcription by altering Nkap intra‐nuclear distribution and obstructing the interaction between Nkap and Hdac3, thereby leading to CHD.

## 1 INTRODUCTION

Congenital heart disease (CHD) is the most common cause of infant mortality.^1^ With the rapid advancements and broad‐scale application of sequencing technology, a portion of CHD caused by genetic alterations has been identified through preconception screening and prenatal diagnosis. This helps reduce the incidence of infants born with CHD, thereby alleviating the associated social burden.^2^, ^3^

*NKAP*, encoding the NF kappa B‐activating protein, is a highly conserved and widely expressed nucleoplasmic protein that plays critical roles in multiple biological processes, including transcriptional repressor, immune cells' proliferation and differentiation, RNA splicing and processing, and promoting survival of adult haematopoietic stem cells.^4^, ^5^, ^6^, ^7^, ^8^ In 2019, Fiordaliso et al. reported for the first time that *NKAP* mutations in the C‐terminal region, including p.R330C, p.R330H, p.R333Q, p.I337T and p.R361Q, were associated with MRXSHD.^9^ MRXSHD is characterized by global developmental delay with hypotonia, delayed speech and mildly delayed walking, often accompanied by somatic marfanoid features. Some patients may also present with cardiac defects.^9^ However, only 10 affected individuals have been reported to date. Therefore, further confirmation is required regarding the association between *NKAP* mutations and MRXSHD, as well as the types of *NKAP* mutations and the phenotypic spectrum they contribute to.

A transcriptome analysis conducted on patient‐derived lymphoblastoid cell lines carrying *NKAP* p.R333Q or p.R361Q mutations revealed the transcriptional dysregulation in multiple biological process pathways, including the Notch signalling pathways.^9^ Despite these findings, the mechanism underlying how *NKAP* mutations manifest in the symptoms of MRXSHD, especially the cardiac malformations, remains unclear and requires further investigation.

In this study, we identified a maternal hemizygote mutation, *NKAP* c.988C>T (p.R330C), in two Chinese foetuses diagnosed with CHD for the first time. We hypothesized that *NKAP* p.R330C was associated with the CHD phenotype observed in these foetuses. Further, we explored the role this mutation played in the pathogenesis of CHD.

## 2 MATERIALS AND METHODS

### 2.1 Patient

In November 2021, a 29‐year‐old woman was admitted to Jinhua Maternal and Child Health Care Hospital due to her unborn child presenting with thickened nuchal translucency. An ultrasound screening revealed the presence of CHD in the foetus, prompting an immediate cytological and molecular evaluation. This study was approved by the ethics committee of Jinhua Maternal & Child Health Care Hospital, Zhejiang Province, China (Approval No. 2020‐4‐068‐2020KY003). Informed consent was obtained from the parents for their inclusion in the study.

### 2.2 Whole exome sequencing and Sanger sequencing

The DNA of the parents and foetus was extracted from the peripheral blood and abortion tissue using the QIAamp DNA Mini Kit (Qiagen, Germany). Trio‐WES was performed using Agilent SureSelect XT Human All Exon V6 Kit (Agilent Technologies, USA) and Illumina Hiseq 2500 system (Illumina, USA). Sequence analysis was conducted using NextGENe software (SoftGenetics LLC, USA). Sanger sequencing was performed to validate the identified mutations. The sequences for the primers involved in this process are listed in Table 1.

**TABLE 1:** Primers sequence.

	Primers sequence
*NAKP* (Sanger)	F: 5′‐TGTAAAACGACGGCCAGTTGGGGATTTTTGGTGCTCTC‐3′ R: 5′‐CAGGAAACAGCTATGACCGAAACTGAGCTTAATGCCAACAAC‐3′
*NKAP* (qRT‐PCR)	F: 5′‐GTTCGTCGAAGAGTCCGAAG‐3′ R: 5′‐GACTGACGAGGAGGCAGAAG‐3′
*DNAH11*	F: 5′‐CTCAAGGGACTGTGGGATGT‐3′ R: 5′‐GGCAGGGCTCTGTAACTCTG‐3′
*DHRS3*	F: 5′‐CATGGGAAGAGCCTAATGGA‐3′ R: 5′‐GACGCTTTGGATGTGCAGTA‐3′
*JAG1*	F: 5′‐GACTCATCAGCCGTGTCTCA‐3′ R: 5′‐TGGGGAACACTCACACTCAA‐3′

Abbreviations: F, Forward primers sequence; R, Reverse primers sequence.

### 2.3 Cell culture and transfection

The HEK293T and HeLa cells were cultured in DMEM medium (Gibco, USA), supplemented with 10% foetal bovine serum (Gibco, USA) and maintained in 5% CO_2_ at 37°C. Wild‐type or mutant GFP‐*NKAP* plasmid DNA, with or without FLAG‐*HDAC3* plasmid DNA (SimGen, China), was transfected into the cells using Lipo2000 following the manufacturer's instructions. After 48 h of culture, the cells were collected for further analysis.

### 2.4 Expression analysis

HEK293T cells transfected with wild‐type or mutant GFP‐*NKAP* were cultured for 48 hours and washed with PBS. Total RNA was collected using TRIzol (TaKaRa, Japan), followed by DNA digestion and cDNA synthesis (YEASEN, China) of *NKAP*, *DNAH11*, *DHRS3* and *JAG1*. Quantitative PCR (qPCR) was then applied for mRNA expression analysis. The primes are listed in Table 1.

For Nkap protein level analysis, the collected HEK293T cells were lysed using RIPA buffer to extract total protein. The protein concentration was measured using the BSA protein assay. Protein samples were then heated with 2*SDS sample buffer at 100°C for 10 min. The samples were separated using SDS‐PAGE, and the Nkap protein was detected by Western blotting (WB).

### 2.5 Subcellular localization of Nkap

HeLa cells were seeded to reach 40%–60% confluence in 6‐well plates with coverslips (Thermo Fisher Scientific, USA) and transfected with FLAG‐*NKAP* plasmids for 36 h. The cells were fixed in 4% paraformaldehyde for 30 min at 4°C and washed three times with phosphate‐buffered saline (PBS). Next, the fixed cells were incubated in a blocking solution containing 1% bovine serum albumin (Sigma‐Aldrich, USA) and 1% normal goat serum (Jackson ImmunoResearch, USA) in PBS with 0.1% Triton X‐100 for 30 min at room temperature. The cells were then incubated with FLAG antibodies (Sigma‐Aldrich, USA) overnight at 4°C. Then, the cells were washed three times with PBS and incubated with secondary antibodies conjugated to Alexa Fluor 488 (Thermo Fisher Scientific, USA) for 1 h at room temperature. The nuclei were stained with DAPI (Sigma‐Aldrich, USA). Images were acquired with fluorescence microscopy.

### 2.6 Co‐immunoprecipitation analysis

HEK293T cells transfected with GFP‐*NKAP* and FLAG‐*HDAC3* were grown for 48 h and washed with PBS. The cells were then lysed with ice‐cold IP lysis buffer containing protease inhibitors for 30 min on ice, followed by centrifuging at 12,000*g* for 30 min at 4°C. The supernatant was incubated with 1 μg of FLAG antibody (Sigma‐Aldrich, USA) overnight at 4°C, followed by incubation with protein‐A agarose beads at 4°C for 2 h. After centrifugation at 1200 rpm for 3 min, the beads were washed three times with 200 μL lysis buffer, and the immunoprecipitation complex was heated with 30 μL 2*SDS sample buffer at 100°C for 10 min. The samples were then separated using SDS‐PAGE and detected by WB.

### 2.7 Statistical analysis

Data are reported as the mean ± SD. The mRNA expression analysis between the two groups was carried out using the Student *t*‐test. *p* < 0.05 was considered statistically significant. Western blot bands and CO‐IP bands analysis were analysed using Image J software. GraphPad Prism 9.3 was used for all statistical analyses.

## 3 RESULTS

### 3.1 *NKAP* p.R330C mutation was identified in two foetuses with CHD

**FIGURE 1:**
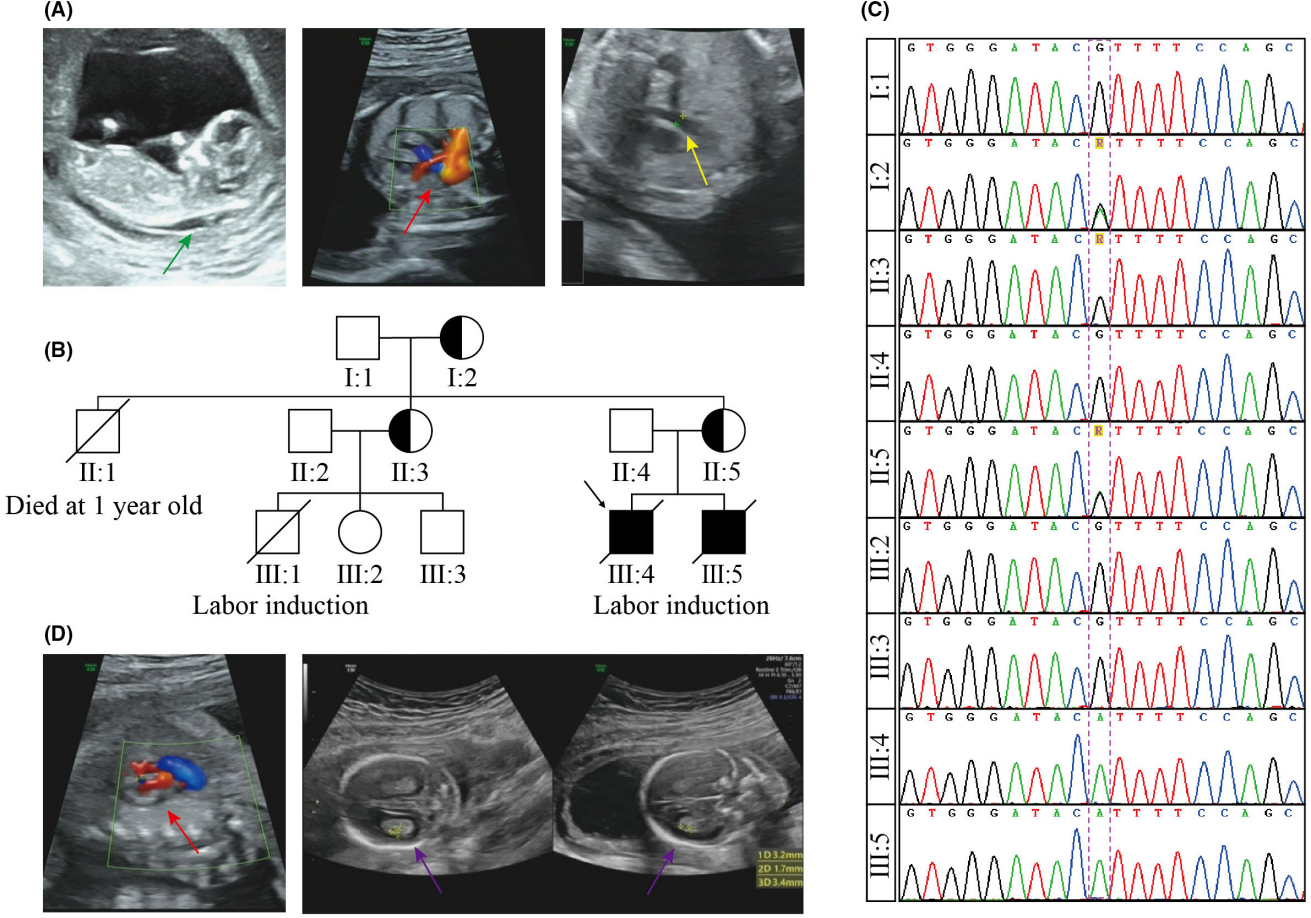
Identification of *NKAP* p.R330C mutation. (A) Prenatal ultrasound images of the proband (III:4). The green arrow delineates a thickened nuchal translucency (NT = 3.0 mm). The red arrow indicates ventricular septal defect. The yellow arrow indicates pulmonary stenosis (diameter = 2.6 mm). (B) Family pedigree and genotype assessment. Squares and circles represent males and females, respectively. Filled symbols indicate affected individuals carrying the *NKAP* p.R330C mutation. Half‐filled symbols represent individuals carrying the *NKAP* p.R330C mutation who consider themselves in perfect health. Open symbols represent individuals who do not carry the mutation or refuse genetic testing. Crossed‐out symbols represent deceased individuals or terminated pregnancies. The proband is indicated by the black arrow. (C) Confirmation of *NKAP* genotype in family members using Sanger sequencing. The purple box indicates the *NKAP* c.988C>T (p.R330C) mutation. (D) Prenatal ultrasound images of the second foetus (III:5). The red arrow indicates ventricular septal defect. The purple arrow indicates choroid plexus cysts.

The proband was a male foetus of Chinese Han descent. At 12 weeks gestation, ultrasound exhibited a thickened nuchal translucency (NT = 3.0 mm) (Figure 1A). However, subsequent karyotyping and chromosomal microarray analysis (CMA) conducted on the amniotic fluid at 18 weeks gestation did not report any abnormalities (data not shown). Pregnancy continued until 24 weeks gestation, and an echocardiogram revealed the presence of CHD in the foetus. Cardiac manifestations included ventricular septal defect, pulmonary stenosis (diameter = 2.6 mm) and overriding aorta, which indicated the possibility of Tetralogy of Fallot (Figure 1A). The foetus also had a mild enlargement of the posterior horns of the lateral ventricles on the left side (left = 10.9 mm, right = 9.4 mm). Before pregnancy termination, all foetal growth parameters were within the normal range. The obstetrician did not report any abnormalities in the appearance of the aborted foetus.

The mother of the proband was 29 years old with a G1P0 obstetric history. She reported no history of illness, medication usage or exposure to either radiation or harmful substances during her pregnancy. Pedigree investigation (Figure 1B) revealed that the mother's elder sister (II‐3) had previously undergone an abortion following the diagnosis of CHD in her male foetus (III‐1). However, she had two other children (III‐2 and III‐3) who were reported to be healthy. The mother's elder brother (II‐1), experienced developmental delay and spinal instability, which rendered him unable to stand or sit independently, and tragically died at 1 year old. The mother confirmed that apart from these instances, no other cardiac disease had been reported in her family.

Trio‐WES was carried out on the aborted tissue and the biological parents. The results indicated a maternal hemizygote missense mutation in the *NKAP* gene, NM_024528.4: c.988C>T, NP_078804.2: p.Arg330Cys (Table 2). In addition, Sanger sequencing revealed that the proband's aunt (II‐3) and grandmother (I‐2) were also carriers of this mutation, while the grandfather (I‐1), father (II‐4) and two cousins (III‐2 and III‐3) did not carry the mutation (Figure 1C). Given its known involvement in cardiac malformations, *NKAP* p.R330C was considered to be the cause of the proband's CHD. The proband was suspected to be a patient of MRXSHD.

**TABLE 2:** Database information and pathogenicity predictions of *NKAP* mutation.

NM_024528.4 (*NKAP*):c.988C > T (p.Arg330Cys)	
Chromosome position	NC_000023.10:g.119064064G>A
Source	Maternal (Het)
ClinVar	Uncertain significance^a^
Allele frequency	/
Publications	Fiordaliso et al.^9^
SIFT	0 (Damaging)
Polyphen2 HVAR	0.861 (Possibly damaging)
Mutation Taster	1 (Disease_causing)
CADD	4.651
REVEL	0.675

^a^ The mutation was index in ClinVar database with a Variation ID 827654.

During the second pregnancy, the mother conceived another male foetus exhibiting a ventricular septal defect for the second time (Figure 1D). Additional abnormalities, including mild tachycardia (167 bpm) and choroid plexus cysts, were also observed during prenatal care (Figure 1D). Prenatal diagnosis confirmed that the foetus carried the same *NKAP* p.R330C mutation.

The *NKAP* p.R330C is absent in healthy groups such as GnomAD database (PM2_Supporting). However, it has been identified in a patient diagnosed with CHD and MRXSHD, where it occurred de novo (PS2).^9^ Another amino acid variation at the same position, *NKAP* p.R330H, has been identified in another patient with MRXSHD appearing de novo and was classified as likely pathogenic (PM5_Supporting).^9^ In our study, the same mutation, *NKAP* p.R330C, was identified in two male foetuses with CHD, whereas it was not detected in healthy male family members (PP1_Supporting). According to the ACMG guideline, the *NKAP* p.R330C mutation was classified as likely pathogenic (PS2+ PM2_Supporting+ PM5_Supporting+ PP1_Supporting).

### 3.2 *NKAP* p.R330C mutation downregulated *NKAP* transcription

**FIGURE 2:**
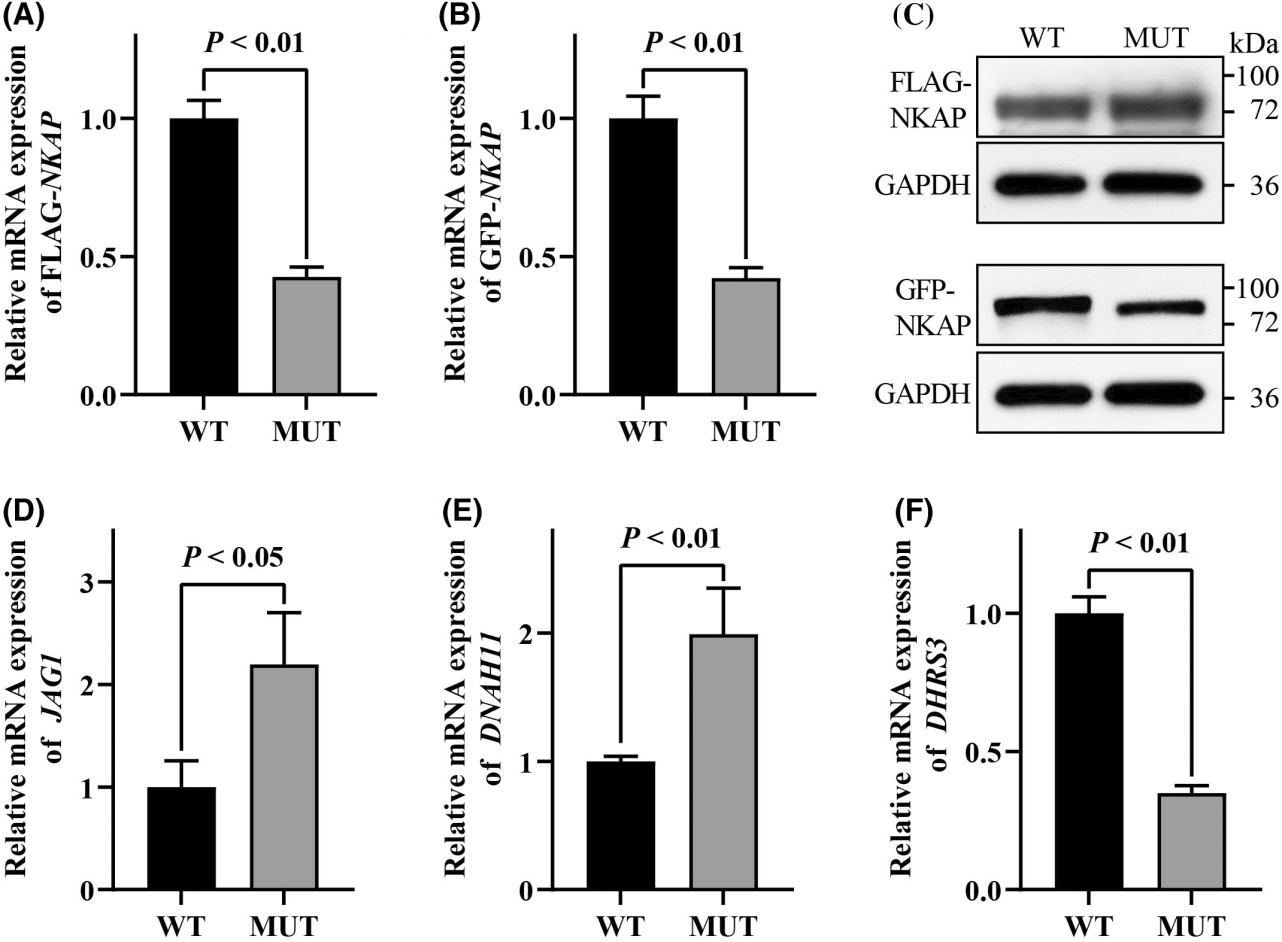
Impact of the *NKAP* p.R330C mutation on gene expression. (A, B) The mRNA expression of both wild‐type and mutated *NKAP* was analysis in HEK293T cells using qRT‐PCR. WT, wild type. MUT, mutant type. (C) Western blot was used to evaluate the expression of wild‐type and mutant Nkap protein levels within HEK293T cells. (D–F) The mRNA expression of cardiac development‐associated genes, *JAG1*, *DNAH11* and *DHRS3*, was analysis by qRT‐PCR.

To evaluate the effect of the p.R330C mutation on *NKAP* expression, we established HEK293T cell lines transfected with either FLAG or GFP targeted *NKAP* in its wild‐type form (*NKAP*‐WT) or its mutant form carrying the p.R330C mutation (*NKAP*‐R330C). Both the mRNA and protein concentrations of *NKAP* were analysed. In HEK293T cells transfected with *NKAP*‐R330C, the *NKAP* mRNA significantly decreased compared to cells transfected with *NKAP*‐WT (Figure 2A,B). However, the total Nkap protein in *NKAP*‐R330C cells showed only a slight decrease or retained equivalent levels (Figure 2C). Unexpectedly, the anti‐Nkap antibody failed to detect bands at the estimated molecular weights of ~48 kDa or ~72 kDa for FLAG‐Nkap protein and GFP‐Nkap protein, respectively. Instead, bands were detected around 72 kDa and 90 kDa for FLAG‐Nkap and GFP‐Nkap, respectively. These could be attributed to post‐translational modification of Nkap, such as SUMOylation.^10^ Taken together, these results suggested that the p.R330C mutation might affect *NKAP* expression.

### 3.3 *NKAP* p.R330C mutation dysregulated the transcription of genes associated with cardiac development

Given *NKAP*'s diverse roles in biological processes, we then investigated whether the *NKAP* p.R330C mutation affected global genome transcription. According to the previously reported transcriptomics data of patients‐derived lymphoblastoid cell line *NKAP*‐R333Q versus *NKAP*‐WT, we identified three genes associated with cardiac septum morphogenesis, *DHRS3*, *DNAH11* and *JAG1*, were significantly upregulated. To investigate the impact of *NKAP* p.R330C mutation on these genes transcription, qPCR was performed in the HEK293T cell line transfected with either *NKAP*‐R330C or *NKAP*‐WT. Both *DNAH11* and *JAG1* showed significant upregulation, while *DHRS3* was downregulated in cells transfected with *NKAP*‐R330C compared to *NKAP*‐WT. This suggests that the upregulation of *DNAH11* and *JAG1* may have a more crucial impact in CHD patients with *NKAP* mutation (Figure 2D–F). Overall, these findings indicated that the *NKAP* p.R330C mutation may contribute to the occurrence of CHD by affecting the gene expression associated with cardiac morphogenesis.

### 3.4 *NKAP* p.R330C mutation blocked Nkap protein binding to the Hdac3 protein

**FIGURE 3:**
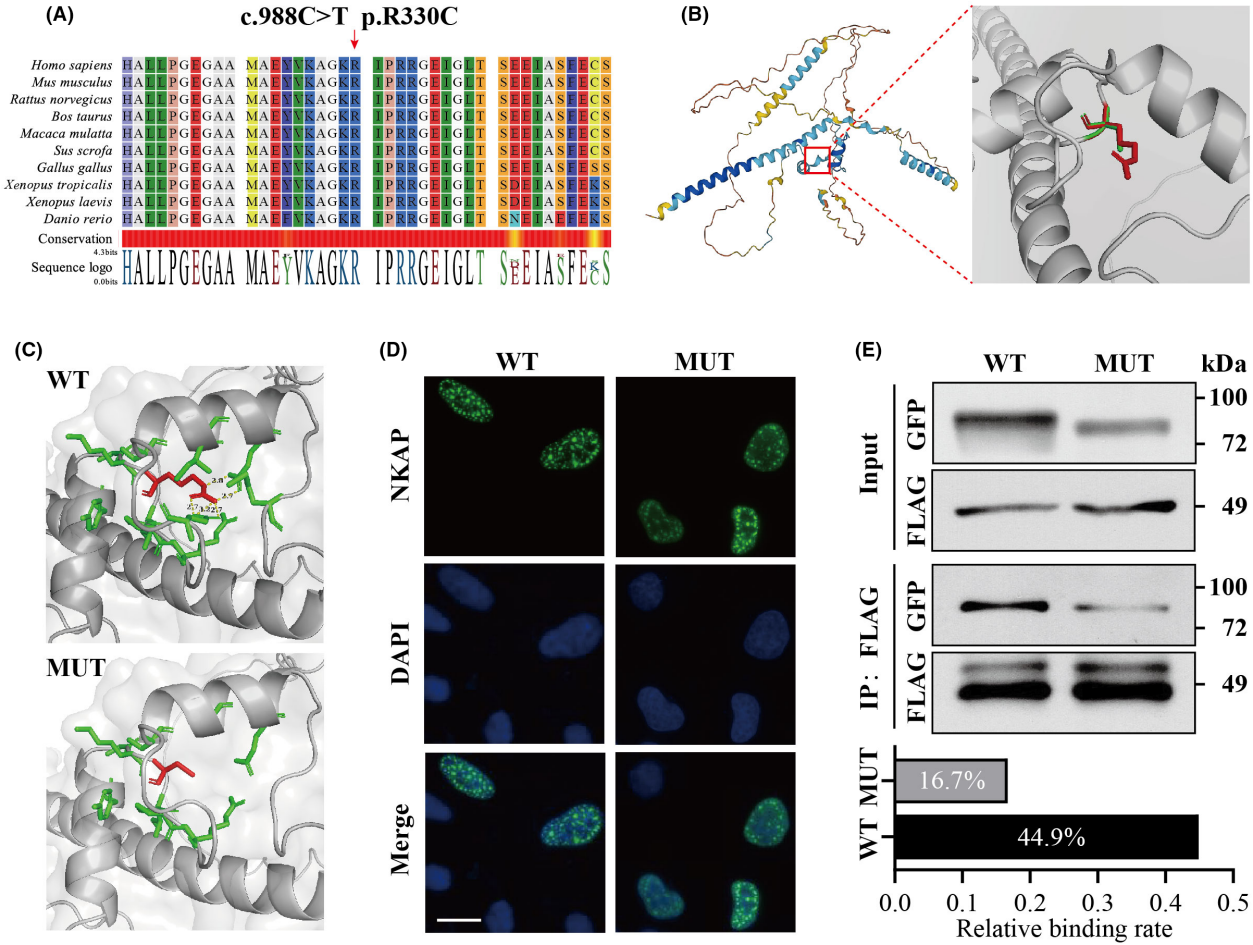
Impact of the *NKAP* p.R330C mutation on Nkap protein structure and Hdac3 protein binding. (A) Evolutionary conservation of amino acid position p.R330C in the Nkap protein. (B) 3D structures of wild‐type and p.R330C mutant Nkap protein. Red and green amino acids represent Arginine and Cystine, respectively. (C) Disruption of hydrogen bonds at amino acid position p.R330 by the *NKAP* p.R330C Mutation. WT, wide type. MUT, mutant type. (D) Subcellular location of FLAG‐tagged wild‐type and mutant Nkap protein. Nucleolar loci are stained with DAPI. Scale bar: 20 μm. (E) CO‐IP analysis of HEK293T cells co‐expressing GFP‐*NKAP* and FLAG‐*HDAC3* to evaluate the effect of *NKAP* p.R330C mutation on Nkap binding to Hdac3. The relative binding rate of Nkap and Hdac3 was assessed.

*NKAP* plays a critical role in transcription and maintaining genome integrity by forming a protein complex with Hdac3. To clarify how *NKAP* p. R330C affects the expression of cardiac development‐associated genes, we next investigated the effect of p.R330C mutation on Nkap's structure, subcellular localization and interaction with Hdac3. Evolutionary conservation showed that the R330 site is highly conserved among various species (Figure 3A). Given the absence of a Protein Data Bank (PDB) file for Nkap based on experimental data, we built a 3D mutant structure using SWISS‐MODEL based on the predicted PDB file from Alphafold (Figure 3B). 3D modelling suggested that the *NKAP* p.R330C mutation disrupts the existing hydrogen bonds at the R330 site, which may affect the structure and stability of Nkap protein (Figure 3C).

Both FLAG‐tagged wild‐type and mutant Nkap protein were observed to be distributed solely in the nucleus (Figure 3D). However, *NKAP*‐WT was evenly distributed throughout the nucleus, whereas the *NKAP*‐R330C was primarily concentrated around the nuclear membrane. This indicates a potential impact on Nkap's ability to regulate transcription due to altered intra‐nuclear distribution. Nkap regulates transcription by binding to Hdac3, while R330C mutation locates in the Nkap C‐terminal region where Nkap interacts with Hdac3. To explore the effect of *NKAP* p.R330C mutation on Nkap binding to Hdac3, HEK293T cell line co‐expressing GFP‐*NKAP* and FLAG‐*HDAC3* were established. CO‐IP showed that Nkap could be successfully pulled down by an anti‐FLAG antibody, which further confirmed the direct interaction between Nkap and Hdac3 (Figure 3E). Intriguingly, there was a noticeable decrease in the pulled‐down Nkap protein in *NKAP*‐R330C compared to the *NKAP*‐WT, reflecting a diminished binding rate between mutant Nkap protein and Hdac3 (a decline from 44.9% to 16.7%). These results revealed that while the p.R330C mutation had little effect on Nkap nuclear translocation, it significantly disrupts Nkap's intra‐nuclear and binding between Nkap and Hdac3, which may further dysregulate the expression of genes associated with cardiac developmental.

## 4 DISCUSSION

In the present study, we identified *NKAP* p.R330C missense mutation in a Chinese family with two male foetuses having CHD and prompted its reclassification as likely pathogenic. No other pathogenic mutations were identified in known CHD‐related genes, indicating that *NKAP* p.R330C is the cause of CHD in this family and both aborted foetuses were potential MRXSHD patients. We found that *NKAP* p.R330C mutation led to a decrease in *NKAP* transcription, while it had little effect on the Nkap protein levels. We also found that *NKAP* p.R330C mutation dysregulated the transcription of genes associated with cardiac development. This disruption primarily occurred due to the distribution of Nkap within the nucleus and an impediment of Nkap and Hdac3 binding.

**FIGURE 4:**
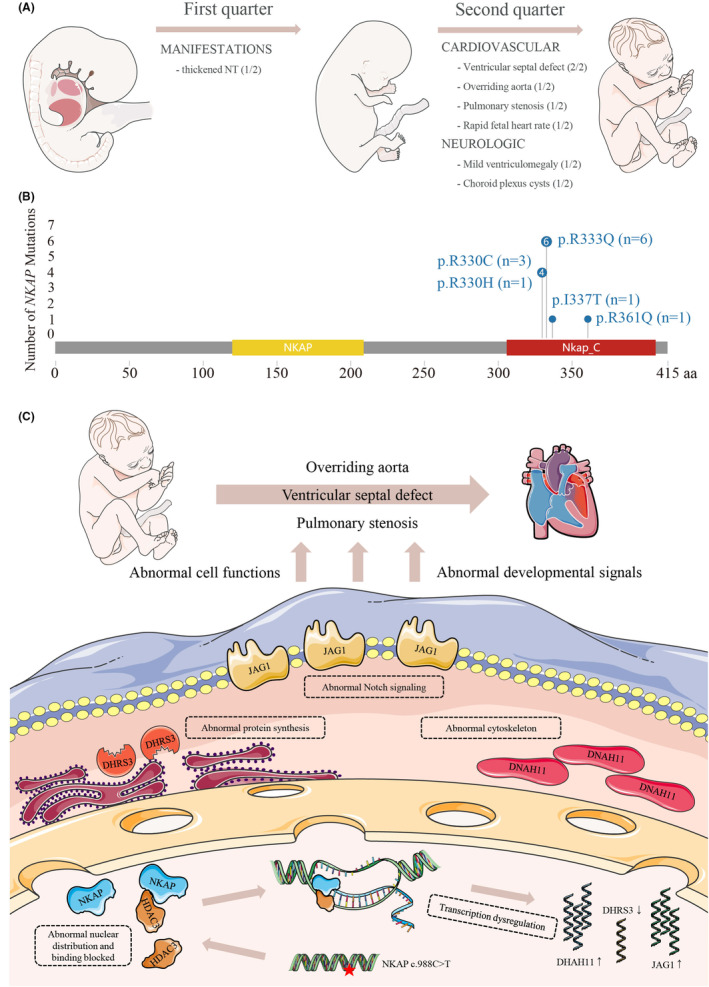
Prenatal phenotypic spectrum and mutation profile of *NKAP*, and the potential mechanism of *NKAP* mutation‐associated CHD. (A) Prenatal phenotypic spectrum of *NKAP* mutation. (B) Mutation spectrum of *NKAP* in 12 patients. (C) The potential mechanism of abnormal cardiac development induced by the *NKAP* p.R330C mutation.

To our consideration, this is the first description of antenatal cases with *NKAP* mutation and the first report of *NKAP* mutation in the Chinese population. Our research significantly contributes to confirming the association of the *NKAP* mutation with CHD. Additionally, we also upgraded the classification of *NKAP* p.R330C from previous ‘variant of uncertain significance’ to ‘likely pathogenic’. Furthermore, we have also contributed to the expansion of prenatal phenotypic spectrum for *NKAP* mutation‐associated developmental disorder. These prenatal anomalies include cardiac defect and thickened NT, which could be observed as early as 12 weeks gestation (Figure 4A).

Until now, all *NKAP* mutations identified in MRXSHD patients were missense: R330C, R330H, R333Q, I337T and R361Q (Figure 4B).^9^ Among them, a majority of the patients (6/10) had de novo mutations. Notably, six individuals from four unrelated families had the same mutation, *NKAP* p.R333Q, while two Asian individuals (Japanese and Korean) had variations in the same amino acid position: *NKAP* p.R330C and p.R330H. Our proband also identified the same mutation *NKAP* p.R330C, suggesting that *NKAP* may have mutation hotspot sites in the C‐terminal region, such as p.R333 and p.R330. It has been reported that loss‐of‐function *NKAP* mutations were detrimental to health and development.^9^, ^11^ However, further research is needed to determine whether missense mutations at these sites impact the functionality of the Nkap protein.

Upon comparing our reported family with previous cases, we observed significant heterogeneity in *NKAP* mutation‐associated cardiac deformities (Table 3). Most individuals (8/12), including two foetuses from our proband's family, had cardiac defects.^9^, ^12^, ^13^ Among them, heart valve disease (4/12) and ventricular or atrial septal defect (4/12) were the two most common conditions, followed by aortic deformations (3/12), patent ductus arteriosus (2/12), pulmonary artery stenosis (1/12) and mild tachycardia (1/12). The cardiac deformation characteristics ranged from mild mitral valve regurgitation to severe Tetralogy of Fallot‐like symptoms. Particularly, all four individuals carrying mutation at p.R330 had cardiac defects, strongly suggesting a correlation between *NKAP* p.R330 mutation and cardiac development. Further investigation is required to provide a comprehensive feature spectrum of NKAP mutation‐associated cardiac deformities.

**TABLE 3:** Cardiac deformation characteristics associated with *NKAP* mutation.

Patients ID		Nucleotide	Protein	Inheritance	Age at evaluation	Cardiac deformities
This study	III:4	c.988C>T	p.R330C	Maternal	24 weeks gestation	Ventricular septal defect, pulmonary stenosis and overriding aorta
	III:5				22 weeks gestation	Ventricular septal defect, mild tachycardia, patent ductal arteriosus, mild semilunar valves stenosis and mild aortic coarctation
Previous studies^9^, ^12^, ^13^	Subject 1	c.988C>T	p.R330C	De novo	10 y	Atrial septal defect
	Subject 2	c.989G>A	p.R330H	De novo	6 y	Small patent ductal arteriosus
	Subject 3	c.988G>A	p.R333Q	Unknown	18 y	Mild mitral valve regurgitation
	Subject 4				10 y	Mitral valve prolapses, mitral regurgitation and aortic root dilatation
	Subject 5	c.988G>A	p.R333Q	Maternal	11 y	Not reported
	Subject 6				5 y	Not reported
	Subject 7	c.988G>A	p.R333Q	Maternal	21 y	Mitral valves prolapse with the minimal mitral regurgitation
	Subject 8	c.998G>A	p.R333Q	Unknown	7 y	History of ventricular septal defect
	Subject 9	c.1010T>C	p.I337T	De novo	10 y	Not reported
	Subject 10	c.1082G>A	p.R361Q	Unknown	16 y	Not reported

Abbreviation: y, years old.

Interestingly, the levels of Nkap protein did not show a significant change, despite the downregulation of its mRNA. This may be attributed to the blocking of Nkap protein ubiquitination degradation in *NKAP* mutant cells. However, the mRNA levels of two cardiac septum morphogenesis‐related genes, *DNAH11* and *JAG1*, were significantly upregulated in cells carrying *NKAP* p.R330C mutation. In contrast, *DHRS3* showed a downregulation, which contradicted the previous report.^9^ It could possibly be the reason for clinical heterogeneity as some individuals, carrying *NKAP* p.R333Q mutation and included in the transcriptome sequencing, did not exhibit cardiac abnormalities.

Heterozygous mutations in *JAG1* have been associated with human cardiac malformations, such as Tetralogy of Fallot (MIM: #187500) and Alagille syndrome (MIM: #118450).^14^, ^15^, ^16^ *JAG1* serves as a cell surface ligand of the *NOTCH* signal, playing critical roles in cardiomyocyte differentiation, cardiac chamber formation and valve morphogenesis.^17^ Research has shown that *JAG1*‐*NOTCH* signalling upregulates the synthesis of collagen‐related proteins, including those involved in the epithelial‐mesenchyme transition (EMT) and extracellular matrix (ECM) deposition in mouse embryonic endocardial cells. In contrast, *DLL4*‐*NOTCH* signalling mainly contributes to the production of TGFβ2, intercellular junction proteins and cytoskeletal proteins.^18^ Therefore, dysregulation of *NOTCH* signalling, such as an elevation in *JAG1* levels, may lead to disorganized cardiac tissue development and ultimately result in congenital heart disease.^19^ *DNAH11*, encoding a component of cilia and flagella known as axonemal outer dynein arm heavy chain, is one of the casual genes of human primary ciliary dyskinesia (MIM: #611884).^20^, ^21^ Recent studies have identified *DNAH11* compound heterozygous or homozygous mutations in heterotaxy patients with CHD.^22^, ^23^, ^24^ Similarly, mice harbouring a homozygous mutation in Dnahc11 gene (the homologous gene of human *DNAH11*) showed a slight increase in Dnah11 expression and exhibited atrioventricular septal defects.^25^ However, this does not appear to be connected with the induction of cardiac malformations through second heart field (SHF) Hedgehog (Hh) signalling.^25^ *DHRS3* encodes retinaldehyde reductase that catalyses the formation of all‐trans‐retinoic acid (ATRA).^26^ Research has shown that *Dhrs3*−/− embryos exhibit ventricular septation defect and late gestation/perinatal lethality in mice.^27^ The ablation of Dhrs3 protein leads to an increased level of ATRA, resulting in the dysfunction of retinoic acid (RA) signalling that regulates the development of multiple organs, including the heart, during embryogenesis.^28^ Additionally, animal models and patients with ATRA or retinol deficiency or excess have also reported cardiovascular defects,^29^, ^30^ further confirming the critical role of *DHRS3* in human cardiac development. Summarily, *JAG1*, *DNAH11* and *DHRS3* are intricately associated with human cardiac development. Nevertheless, the molecular mechanisms underlying how dysregulated expression of these genes impacts cardiac development require further research. These findings suggested that the *NKAP* p.R330C mutation might affect cardiac development by regulating the expression dosage of *JAG1*, *DNAH11* and *DHRS3*. *DNAH11* and *DHRS3* are potential pathogenic genes for CHD.

Nkap protein participates in various biological processes in association with the Hdac3 protein.^8^, ^31^, ^32^ The *NKAP* p.R330C, located in exon 8, modifies a highly conserved amino acid in the C‐terminal of Nkap protein. 3D modelling suggested that the mutation destroys the hydrogen bond at p.R330, potentially altering the Nkap protein structure and affecting protein–protein interactions. The CO‐IP analysis revealed that the p.R330C mutation blocks the binding of Hdac3 and Nkap protein, which might directly impacting the transcription progress. Furthermore, p.R330C prompts an accumulation of Nkap protein around the nuclear membrane, where transcriptional activity is highly variable.^33^ Combining the dysregulation of gene transcription involved in cardiac development, these findings suggested a potential mechanism for *NKAP* p.R330C‐induced CHD: the mutation alters Nkap protein intra‐nuclear distribution and inhibits its binding to Hdac3 protein, thereby disrupting the transcription of cardiac development‐related genes and ultimately leading to CHD, manifested as ventricular septal defect, overriding aorta and pulmonary stenosis (Figure 4C). This study is the first to reveal the potential pathogenic mechanism of *NKAP* mutation‐induced CHD. Nevertheless, as our patients did not exhibit additional MRXSHD features, further research is required to comprehend the mechanisms contributing to MRXSHD‐related developmental delay, mental retardation and other manifestations.

In summary, we identified the *NKAP*‐R330C mutation in two Chinese foetuses with CHD for the first time, confirming the association between *NKAP* mutation and CHD and suggesting its reclassification as likely pathogenic. We also demonstrated the potential prenatal phenotypic spectrum of individuals carrying *NKAP* mutations. Functional analysis revealed that *NKAP* p.R330C altered the intra‐nuclear distribution of Nkap protein and its binding to Hdac3, leading to dysregulation of cardiac development‐associated gene transcription and ultimately CHD in foetuses. Our results provide novel insights into CHD genetic counselling and prenatal diagnosis and demonstrate the potential pathogenic mechanism of *NKAP* mutation‐associated CHD.

## AUTHOR CONTRIBUTIONS

**Xiayuan Xu:** Conceptualization (lead); funding acquisition (lead); investigation (lead); project administration (lead); writing – original draft (equal). **Chengcheng Gao:** Data curation (lead); visualization (lead); writing – original draft (equal). **Fenglei Ye:** Methodology (equal). **Aohui Peng:** Methodology (equal). **Jianbo Xu:** Investigation (equal); methodology (equal). **Keqin Jin:** Investigation (equal); methodology (equal). **Jun Zhang:** Investigation (equal); methodology (equal). **Yun Ye:** Investigation (equal); methodology (equal). **Yanfen Yang:** Investigation (equal). **Xuan Zhang:** Resources (equal). **Shuangshuang Shen:** Supervision (equal); writing – review and editing (equal). **Fan Jin:** Supervision (lead); writing – review and editing (lead).

## FUNDING INFORMATION

This work was supported by grants from the Jinhua Science and Technology Program (No. 2020‐4‐068).

## CONFLICT OF INTEREST STATEMENT

Chengcheng Gao and Xuan Zhang were employed by Dian Diagnostics Group Co., Ltd. The remaining authors declare that the research was conducted in the absence of any commercial or financial relationships that could be construed as a potential conflict of interest.

## Data Availability

The datasets for this article are not publicly available due to concerns regarding participant/patient anonymity. Requests to access the datasets should be directed to the corresponding authors.
